# Detailed Regulatory Mechanism of the Interaction between ZO-1 PDZ2 and Connexin43 Revealed by MD Simulations

**DOI:** 10.1371/journal.pone.0021527

**Published:** 2011-06-23

**Authors:** Fei Xiao, Jingwei Weng, Kangnian Fan, Wenning Wang

**Affiliations:** 1 Shanghai Key Laboratory of Molecular Catalysis and Innovative Materials, Department of Chemistry, Fudan University, Shanghai, People's Republic of China; 2 Institute of Biomedical Sciences, Fudan University, Shanghai, People's Republic of China; Griffith University, Australia

## Abstract

The gap junction protein connexin43 (Cx43) binds to the second PDZ domain of Zonula occludens-1 (ZO-1) through its C-terminal tail, mediating the regulation of gap junction plaque size and dynamics. Biochemical study demonstrated that the very C-terminal 12 residues of Cx43 are necessary and sufficient for ZO-1 PDZ2 binding and phosphorylation at residues Ser (-9) and Ser (-10) of the peptide can disrupt the association. However, only a crystal structure of ZO-1 PDZ2 in complex with a shorter 9 aa peptide of connexin43 was solved experimentally. Here, the interactions between ZO-1 PDZ2 and the short, long and phosphorylated Cx43 peptides were studied using molecular dynamics (MD) simulations and free energy calculation. The short peptide bound to PDZ2 exhibits large structural variations, while the extension of three upstream residues stabilizes the peptide conformation and enhanced the interaction. Phosphorylation at Ser(-9) significantly weakens the binding and results in conformational flexibility of the peptide. Glu210 of ZO-1 PDZ2 was found to be a key regulatory point in Cx43 binding and phosphorylation induced dissociation.

## Introduction

PDZ (PSD-95/DLG,/ZO-1) domains belong to a group of modules mediating the interactions between proteins [1]–[3]. Hundreds of PDZ domains have been found in each mammalian genome [1], [4]–[9]. A large class of PDZ domain-containing proteins have been identified that mediate targeting and clustering of channels, receptors, cell adhesion proteins, and other signaling enzymes at the specific sites of cell-cell contact, including synapses [10]–[16]. A typical PDZ domain consists of ca. 90 amino acids and has a compact globular fold composed of a core of six β strands (βA–βF) and two α helices (αA, αB), binding the C-terminal motifs of their target proteins, PDZ domains target, cluster and route these proteins [15], [17], [18]. The interactions between PDZ domains and the C-terminal motif of target proteins have been extensively studied and well understood [4], [6], [18], [19]. C-termini of target proteins recognize and bind to a well defined pocket between βB and αB of each PDZ domain [15], [18], [20]–[22]. PDZ domains are grouped into two classes according to ligand sequence. Class I PDZ domains bind to C terminal motifs with the sequence of [Ser/Thr-X-Φ COOH] and class II PDZs bind to the sequence of [Φ-X-Φ-COOH], where Φ is any hydrophobic amino acid and X is any amino acid [23].

**Figure 1:**
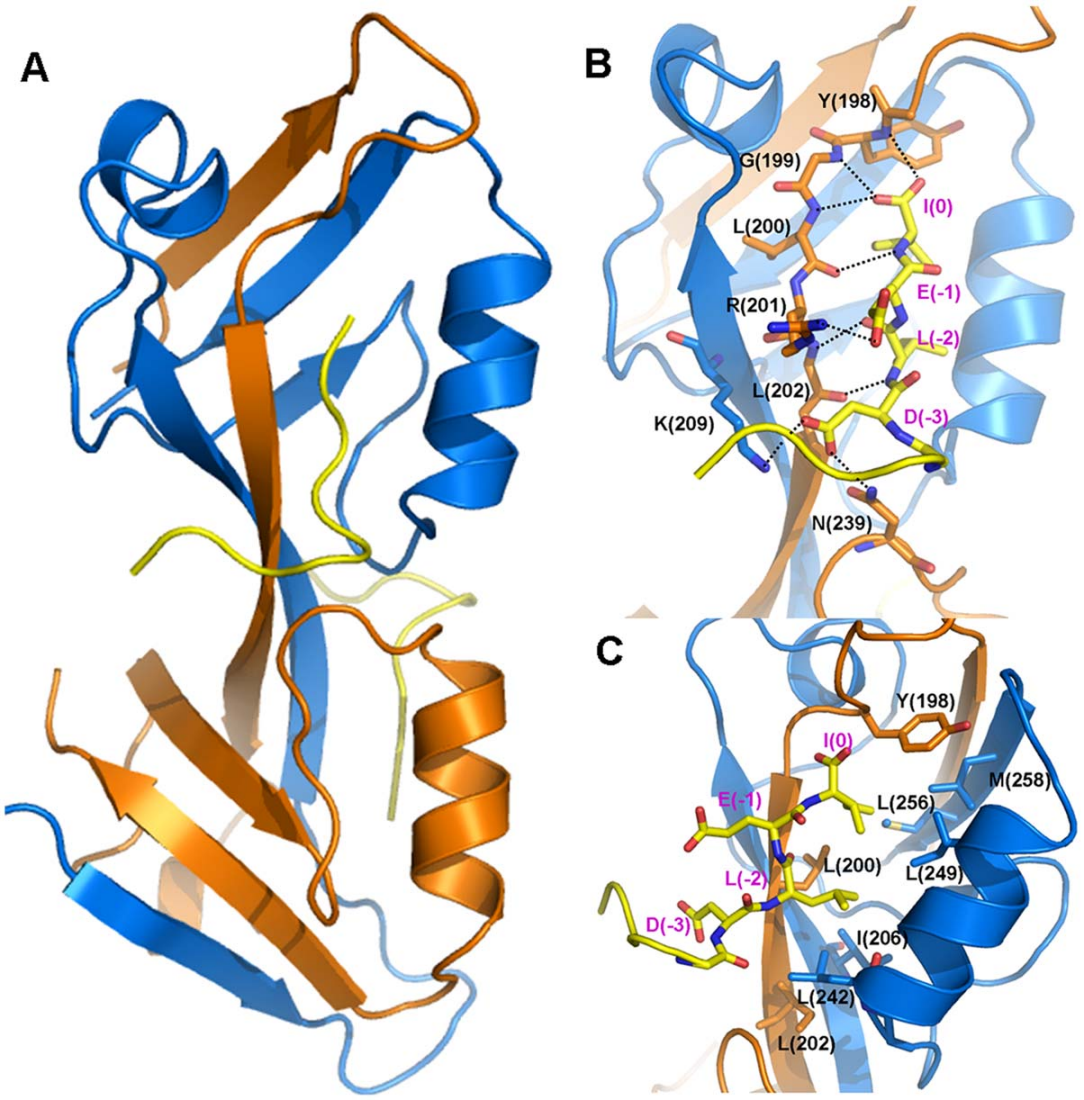
Crystal structure of ZO-1 PDZ2-Cx43 complex. **A**) Ribbon diagram of ZO-1 PDZ2 dimer in complex with 9 aa Cx43 peptide. The two PDZ domains in the dimer are shown in blue and orange respectively, and two Cx43 peptides are in yellow. Detailed polar **B**) and hydrophobic **C**) interactions of the C-terminal fragment of Cx43 peptide with PDZ2.

Zonula occludens-1 (ZO-1) is the first identified member of the PDZ family proteins [24]–[26]. It does not only act as passive scaffold in organizing gap junction (GJ) complexes including connexins and cytoskeletals, but also actively participates in the dynamic remodeling of GJs in a number of cellular systems, including cardiomyocytes, fibroblasts and neurons [27]–[30]. The carboxyl tail of connexin43 (Cx43) binds to the second PDZ domain of ZO-1 (ZO-1-PDZ2) [27], [31], mediating the regulation of GJ plaque size and dynamics in various cell models [28], [30]. Recently, Chen *et al.* solved the crystal structure of ZO-1-PDZ2 in complex with a 9 aa C-terminal peptide of Cx43 (^−8^RPRPDDLEI^0^) [32]. The structure exhibits a domain-swapped dimerization arrangement of PDZ2, each domain interacting with the Cx43 peptide through its canonical ligand-binding pocket (Figure 1A). The binding of Connexin43 to ZO-1 PDZ2 via carboxyl tail belongs to canonical class II PDZ/ligand interaction. In addition to the most C-terminal residues, the upstream residues of the peptide contribute to the binding through charge-charge interactions with an extended ligand binding pocket at the dimer interface of PDZ2 [32]. (Figure 1B, C) Biochemical studies further indicated that a longer peptide of 12 aa (^−11^ASSRPRPDDLEI^0^) has higher binding affinity to PDZ2 and the phosphorylation of the upstream residues Ser (-9) and Ser (-10) remarkably reduced or abolished the peptide binding [32]. These results are consistent with the early findings that Ser(-9) and Ser (-10) are substrates if several kinases, including PKA and Akt [33]–[35], suggesting that these two residues may function as phosphorylation-mediated regulatory switch for the ZO-1/Cx43 interaction.

Due to the important roles of Ser(-9) and Ser(-10), it would be informative to obtain the atomic details of the interactions between PDZ2 and the two serine residues. Unfortunately, the experimental effort of obtaining the crystal structure of PDZ2 in complex with the long peptide failed [32]. For a deeper understanding of the ZO-1 PDZ2-Cx43 interaction and the phosphorylation-mediated regulation mechanism, we performed molecular dynamics (MD) simulations to study the interaction between ZO-1 PDZ2 and the short, long and phosphorylated Cx43 peptides. The simulation results showed that the extended residues in the long peptide significantly enhanced the binding of the N-terminal tail of the peptide to the protein and the phosphorylation at Ser(-9) disrupted the binding of N-terminal region, resulting in large structure variation of the peptide. Glue210 on PDZ2 was found to play an important role in interacting with the two serine residues.

## Results

Eighty ns MD simulations were carried out for ZO-1 PDZ2 in complex with three different Cx43 peptides: 9 aa short peptide (^−8^RPRPDDLEI^0^), 12 aa long peptide (^−11^ASSRPRPDDLEI^0^) and phosphorylated 12 aa peptide at Ser(-9). For each system, two trajectories with different initial velocities were produced, and the trajectories showed high consistencies. For simplicity, most of the following detailed analyses were based on one trajectory for each system.

### The 9 aa short peptide of Cx43 showed large structure variation in binding with ZO-1 PDZ2

**Figure 2:**
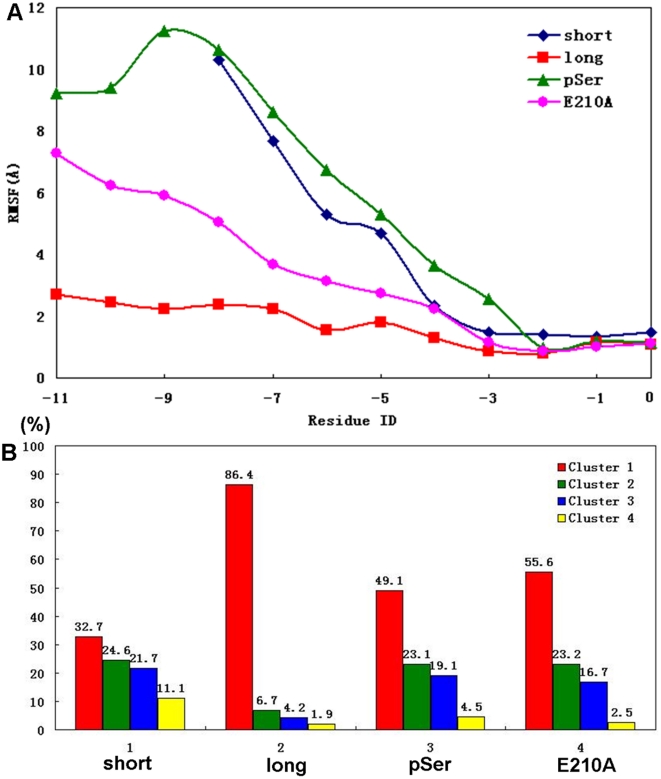
Conformational flexibility of short, long and phosphorylated Cx43 peptide bound to ZO-1 PDZ2. **A**) C-alpha RMSF of the short (dark blue), long (red), E210A long (pink) and phosporylated (green) Cx43 peptide in the MD simulations. **B**) Results of cluster analyses in the short, long, E210A long and phosphorylated peptide systems. Conformational percentages of the four largest clusters are shown.

The binding of the C-terminal parts of the short Cx43 peptides to each PDZ domain are very stable throughout the simulations. As in the typical Class II PDZ domain-peptide complexes, the last three residues of the peptide have extensive polar and hydrophobic interactions with the binding groove between the αB helix and βB strand. The hydrophobic side-chains of Ile(0) and Leu(-2) insert into the hydrophobic pocket formed by Leu242 and Ile249 on αB helix, Leu256 and Met258 on βF strand, Tyr198, Leu200 and Leu202 on βB′ strand, and Ile24 on βC′ strand. The polar interactions include the backbone/side chain hydrogen bonds between Ile(0) and Leu(-2) of the peptide and Tyr198, Gly199, Leu200, Arg201 and Leu202 on βB strand of the PDZ domain, and a salt-bridge between Glu(-1) of the peptide and Arg201 on βB strand of PDZ2 (Figure 1B, 1C). In addition to the last three residues, Asp(-3) of the peptide forms a hydrogen bond with Asn239 and a salt bridge with Lys209. These interactions in the initial crystal structure were preserved along the simulation trajectory and restrained the motion of the C-terminal end. The root-mean-square fluctuation (RMSF) values of the last four residues of the peptide are below 2 Å (Figure 2A).

**Figure 3:**
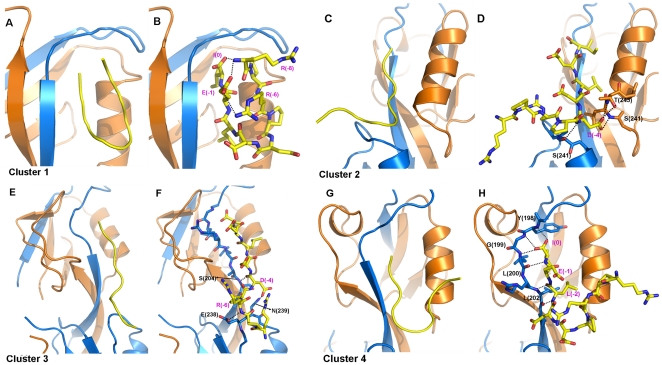
Representative structures of the largest four clusters in the short peptide system. **A**), **C**), **E**), **G**) showing the peptide conformations in ribbon diagrams. **B**), **D**), **F**), **H**) detailed interactions between the Cx43 peptide and PDZ2. Cx43 peptides are shown in stick model. Hydrogen bonds and salt-bridges are denoted with dotted lines.

The N-terminal part of the peptide, however, was much less stable than the C-terminal part during the simulations. It deviated significantly from the initial position in the crystal structure and exhibited large RMSF values of more than 8 Å (Figure 2A). The high flexibility results in sampling diverse conformations of the peptide along the simulation trajectory. To examine the conformational diversity, we performed cluster analysis of the peptide conformations (see Method for details). The peptide conformations could be classified into 31 clusters with an RMSD cutoff of 1.5 Å. The first four largest clusters contain conformations more than 90% of the total (Figure 2B), and have distinct peptide conformations. The largest cluster contains 32.7% of the total conformations and in its representative structure the peptide adopts a hairpin like conformation (Figure 3A). The N-terminal part of the peptide folds back to interact with its C-terminal residues. Two salt-bridges are formed between the positively charged N-terminal amide group and the negatively charged carboxyl group of Ile(0) and/or the side-chain of Glu(-1). The hairpin-like conformation is further stabilized by other intra-peptide interactions, such as the charge-charge and hydrogen bond interactions between Arg(-6) and Glu(-1) (Figure 3B).

In the second largest cluster, the peptide adopts an L-shaped conformation (Figure 3C). The peptide makes a turn at Asp(-4) and the residues N-terminal to it extends along the groove between the two PDZ domains. The side chain of Asp(-4) forms hydrogen bonds with Ser241 and Thr243 on the αB helix. The backbone of Asp(-4) forms hydrogen bond with Ser241 on the αB helix of the opposing PDZ domain. (Figure 3D) These interactions pulled the peptide closer to αB helix and slightly changed the peptide conformation with respect to that in the crystal structure. The residues N-terminal to Asp(-4) do not interact with the protein directly. Overall, the peptide conformation in the second cluster resembles that in the crystal structure, but the interactions between Asp(-4) and the protein are different.

The typical peptide conformation in the third cluster is an extended one, with its N-terminal tail interacting with the opposing PDZ domain (Figure 3E). The backbone carbonyl of Asp(-4) forms hydrogen bond with the amide of Ser204 on the βB strand and the side chain of Asp(-4) forms hydrogen bond with Asn239 on the αB-βE loop (Figure 3F). Another hydrogen bond was formed between the amide group of Arg(-6) and the side chain of Glu238.

The representative conformation of the peptide in the fourth cluster can be described as V-shape (Figure 3G, H). While the C-terminus still binds tightly to the protein, the peptide turns at Asp(-4) and places its N-terminal end close to the C-terminal end. There is no specific interaction between the N-terminal part of the peptide and the PDZ domain, and it is fully exposed to the solvent and fluctuating freely.

The simulations of the short peptide complex showed a sampling of multiple conformations, the majority of which is the above four clusters. The transition between different clusters happened more than once per 50 ps on average during the 80 ns trajectory. The high transition frequency indicates a dynamic binding mode of the short peptide, which is dramatically different from the static picture as the crystal structure showed.

### The 12 aa long peptide of Cx43 binds tightly to ZO-1 PDZ2

**Figure 4:**
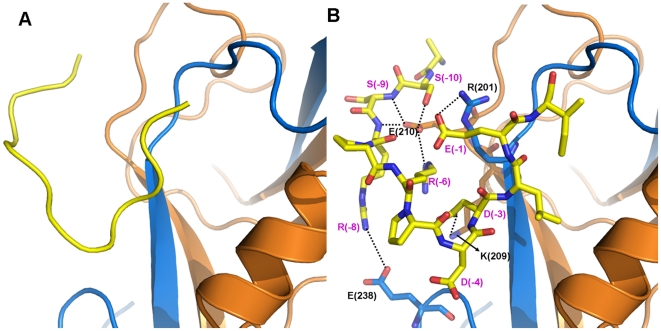
Binding of the long Cx43 peptide to ZO-1 PDZ2. **A**) Ribbon diagram showing the representative structure of the largest cluster of the long peptide conformations bound to ZO-1 PDZ2. **B**) Detailed interactions between the N-terminal fragment of the peptide (A(-11) to D(-3)) and PDZ2.

The C-terminus of the long peptide shares a similar interaction network as observed in the short peptide system. The hydrophilic and hydrophobic interactions between Ile(0), Glu(-1), Leu(-2) and Asp(-3) on the peptide and βB, βC and αB at the binding pocket are extensively interacting with each other throughout the simulation, just like in the short peptide system. Besides the C-terminal region of the peptide, the N-terminal part also contributes to the binding to PDZ domain. The newly added Ser(-10) and Ser(-9) form several hydrogen bonds by their side-chains and main-chains with Glu210 (Figure 4B). The positively charged Arg (-6) and Arg (-8) interact with Glu210 and Glu238 through both electrostatic attraction and hydrogen bonding. The simulation trajectories also showed that the salt bridges between the C-terminal residues (Glu(-1) and Asp(-3)) and the protein were notably enhanced with respect to the short peptide system, probably due to the binding of the N-terminal part. These specific interactions in the N-terminal part of the peptide with the protein evidently reduced the diversity of the peptide conformation. The cluster analysis gave only 10 clusters in this long peptide system, compared with 31 clusters in the short peptide system (Figure 2B). The largest cluster has an overwhelming number of conformations of more than 86%. The peptide adopts an L-shaped conformation very similar with that in the crystal structure, with the N-terminal end of the peptide extending towards Glu210 (Figure 4A). The intra-peptide electrostatic interactions between the side-chains of Arg(-6) and Asp(-4)/Asp(-3) also stabilize the peptide conformation.

To validate the role of E210 in the long peptide binding, we performed another two 80 ns simulations of the long peptide complex by mutating E210 to alanine. The E210A mutation evidently changed the behavior of the N-terminal region of the long peptide bound with ZO-1 PDZ2. Although the interactions between the C-terminal part of the peptide and the protein were hardly changed, the N-terminal region became more flexible than that of the wild-type complex (Figre 2A). Cluster analysis gave 11 clusters of peptide conformations and the sizes of the three largest clusters were 55.6, 23.2 and 16.7 percentages respectively, indicating larger conformational diversity than that of the wild-type complex. In the largest cluster, the peptide kept a similar conformation as that in the wild-type complex. Its C-terminal region was buried in the canonical PDZ binding pocket, and its N-terminal region interacts with the protein through hydrogen bonds Ala210-Arg(-6) and Glu225-Ser(-10). In the second and the third largest clusters, Ala210 was not involved in the peptide-protein interactions. Therefore, the simulation validated the importance of E210 and showed that the E210A mutation changed the peptide binding mode by weakening/disrupting the original Glu210-involved interactions.

### Phosphorylation at Ser(-9) disrupted the binding of the N-terminal region of the long peptide to ZO-1 PDZ2

**Figure 5:**
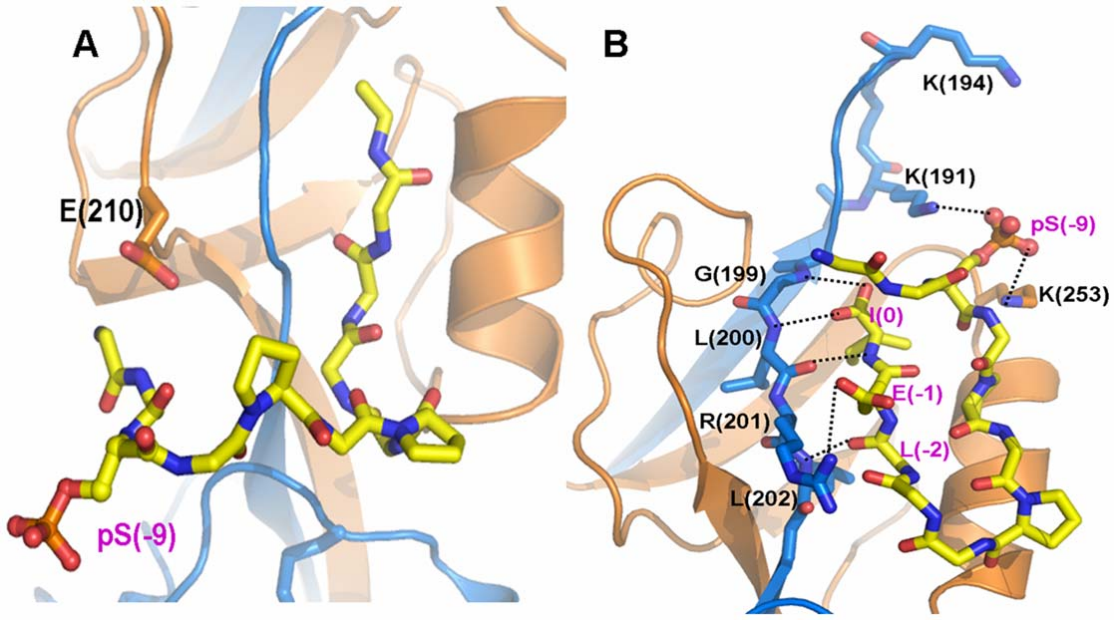
Phosphorylation induced conformational change of the Cx43 peptide bound to ZO-1 PDZ2. **A**) Structure of the phosphorylated peptide in the beginning of the simulation. **B**) Conformation of the phosphorylated peptide at the end of the 80 ns simulation and its detailed interactions with PDZ2.

The simulations of the phosphorylated long peptide system showed that phosphorylation on Ser(-9) does not affect the interaction network between the C-terminal part of the peptide and the protein, but remarkably disturbs the binding of the N-terminal region. The initial structure of the simulation was based on the end structure of the long peptide simulation system by mutating Ser(-9) to pSer(-9) (Figure 5A). Soon after the simulation began, the N-terminal tail of the phosphorylated peptide moved away from the original position at the Glu210-centered region. This is most likely due to the strong electrostatic repulsion between the phosphate group and the negatively charged Glu210. The N-terminal region of the peptide experienced large-scale conformational changes along the trajectories similar with those observed in the short peptide system. (Figure 2A). Cluster analysis gave 16 clusters of peptide conformations, and the three largest clusters contain 49.1%, 23.1% and 19.1% conformations respectively, indicating a different binding mode from that found in the long peptide system (Figure 2B). Similar with the short peptide system, the peptide adopts a hairpin-like conformation in the representative structure of the largest cluster. The phosphate group of Ser(-9) interacts with the positively charged residues Lys191, Lys194, and Lys253 on the protein surface near the C-terminus of the peptide. The peptide made a sharp turn at Asp(-4) and several intra-peptide interactions, including salt-bridges between Arg(-6)-Asp(-4) and Arg(-6)-Asp(-3) and several backbone hydrogen bonds, stabilizing the hairpin conformation. (Figure 5B). Overall, the phosphorylation of Ser(-9) segregated the N-terminal tail of the peptide from the Glu210-centered region, thereby destabilized the peptide conformation in the long peptide system.

To quantitatively evaluate the perturbation of the phosphorylation of Ser(-9) of Cx43 on its binding affinity to PDZ2, we performed the free energy perturbation (FEP) calculation to compare the binding free energy changes between wild type and pSer(-9) long peptide systems. A thermodynamic cycle was designed to calculate the binding free energy difference through “alchemical transformation” on Ser(-9) to pSer(-9) in the free and bound states, respectively (Figure S1). The binding free energy difference upon phosphorylation is thereby equal to:
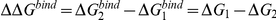



The calculated ΔΔG^bind^ is 8.6±6.7 kcal/mol. This value gives a rough estimate of the binding affinity change upon phosphorylation on Ser(-9). According to the fluorescence-based assay of the binding affinity of Cx43 long peptide to PDZ2, the dissociation constants for wild type peptide is 7.2 µM and that for pSer(-9) was estimated to be >100 µM, resulting in a value of ΔΔG^bind^ >1.57 kcal/mol [32]. Considering the errors in both calculation and experiments, the free energy estimation is generally in agreement with the binding affinity measurement.

### The inter-domain motions of ZO-1 PDZ2

The domain-swapped dimerization is a distinct feature of ZO-1 PDZ2 by tightly linking two PDZ domains together. To examine the possible correlation between the conformational dynamics of PDZ2 dimer and the ligand binding, we analyzed the variations of the inter-domain orientation of PDZ2 along the simulation trajectories. The domain orientation was defined by a vector pointing from the center of mass of each PDZ domain (MC1 or MC2) to the C_α_ atom of His205 located at binding interface of two domains (C_α_205 or C_α_205′). Based on this, the dihedral angle of (MC1-C_α_205-C_α_205′-MC2) was used to describe the relative orientation between the two PDZ domains. As shown in Table S1, the average value of the dihedral angle differs between various systems and between trajectories of the same system, with evident fluctuations. This indicates that there are intrinsic inter-domain conformational movements in ZO-1 PDZ2 dimer, but this conformational dynamics has no obvious correlation with the peptide binding states.

## Discussion

**Figure 6:**
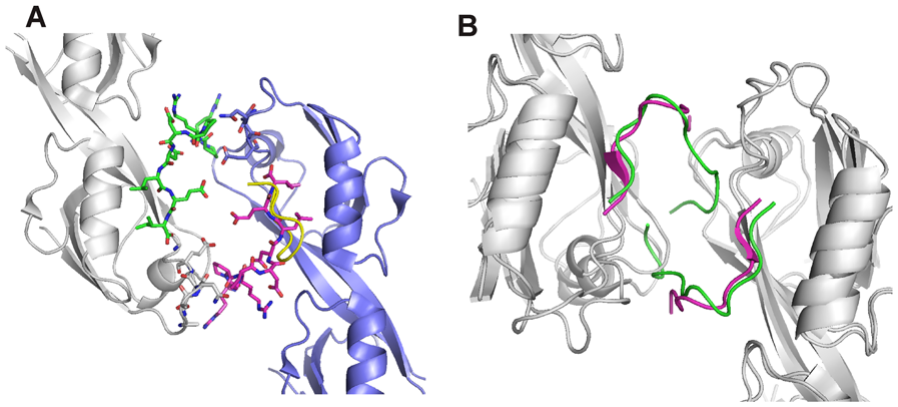
Peptide conformation affects crystal packing. **A**) The packing interface of two PDZ2/Cx43 complexes (blue and white) in adjacent unit cells is shown. The N terminal tails of short peptides (green and magenta) interact with PDZ2 domains (blue and white) in the neighboring unit cells, respectively. The peptide shown in yellow is a representative structure of Cluster 2 in MD simulation of short peptide system. **B**) Superposition of the end structure of the long peptide complex with ZO2/Cx43 crystal structure. Clashes can be seen between the long peptides (green) and PDZ2 domains in the neighboring unit cells, showing that the N terminal tails of the long peptide perturb the crystal packing.

Our simulations provide a detailed picture at atomistic level of the interaction between ZO-1 PDZ2 and the C-terminal tail of connexin43, especially the functional role of the upstream serine residues in regulating the binding. Along the two 80 ns trajectories the 9 aa short peptide bound to PDZ2 in the crystal structure showed large structure variations. The charge-charge interactions involving upstream residues Asp(-4) and Arg(-6) in the crystal structure vanished during the simulation and the N-terminal tail of the peptide was frequently sampling diverse conformations. Structure diversity of the peptide bound to PDZ domain was also found in previous Monte Carlo simulations [36]. The peptide conformation in the crystal structure of ZO-1 PDZ2/Cx43 coincides with the structures in the second cluster of our MD simulations. One might ask why the peptide conformation of the largest cluster in MD simulation did not appear in the crystal structure. This may be attributed to the crystal packing. The Cx43 peptide contributes to the packing interaction with its N terminus interacting with PDZ2 in the neighboring unit cell (Figure 6A). On the contrary, the N terminal tail of Cx43 peptide in Cluster 1 folds back to interact with its C terminus (Figure 6A) and unlikely contributes to the crystal packing. Cluster 2 structures occupy comparable percentage in the structure ensemble with those of Cluster 1, and benefit from the packing interactions. Compared with the crystal structure, the dynamic nature of the N-terminal tail of the short peptide reflects a more realistic picture of the binding to PDZ2, indicating that the charge-charge interactions at the extended binding pocket of PDZ2 are not sufficient to stabilize the peptide conformation.

Early NMR titration study showed that the ZO-1 PDZ2 binding region on Cx43 includes C-terminal 19 residues according to the disappearance or weakening of peaks in the ^1^H-^15^N-HSQC spectrum [37]. The later study quanltitatively measured the binding affinities of PDZ2 with Cx43 C-terminal 9 aa and 12 aa peptides showing that the longer one has stronger binding to PDZ2 and further upstream extension does not increase the affinity [32]. Consistent with this result, our MD simulations showed that the peptide conformation was much more stable in the 12 aa long peptide system, and the overall conformation is similar with that of the short peptide in the crystal structure. Comparison of the structures bound with short and long peptides, however, reveals that extending the peptide by three residues at the N terminus will definitely disturb the crystal packing due to the clashing between the N terminal tail of the long peptide and PDZ2 in the neighboring unit cell (Figure 6B). This perturbation may results in difficulties in crystal growing or remarkable changes in crystal screening conditions. The simulation further revealed that Glu210 of PDZ2 played an important role in enhancing the ligand binding through hydrogen bonding interactions with both Ser(-9) and Ser(-10) on the extended peptide. At the same time, the polar interactions between the C-terminal residues of the peptide with PDZ2 were remarkably strengthened with respect to the short peptide system, further emphasizing the importance of the upstream residues Ser(-9) and Ser(-10).

The phosphorylation of Ser(-9) was shown to severely weaken the ligand binding experimentally [32]. The MD simulation and free energy calculation results conformed with this observation and revealed that the like charge repulsion between Glu210 of PDZ2 and pSer(-9) serves as the driving force for dissociation. Like the case of the short peptide, the N-terminal tail of the phosphorylated peptide exhibited large conformational flexibility. The experiments also found that the phosphomimetic mutation S(-10)E remarkably weakened the peptide binding [32]. This is comprehensive since Ser(-10) also contributes to the interaction with Glu210 through hydrogen bonding, which will be disrupted by electrostatic repulsion between negatively charged Glu(-10) and Glu210. Therefore, Glu210 does not only enhance the peptide binding but also serves as a critical trigger for phosphorylation-induced ligand dissociation.

It is worth noting that the time scale of our simulations may suffer from the insufficient sampling of the conformational spaces of the Cx43 peptides, especially for the long and the phosphorylated peptides since the initial structures in these simulations were not the “native” structures from experiments. However, the distinct behaviors of the three peptides in binding with PDZ2 and the good repeatability of the trajectories suggest that simulation results reflect the nature of the regulatory mechanism of Cx43 binding to ZO-1 PDZ2.

## Methods

### Atomic Coordinates

The initial structure of the short peptide bound ZO-1 PDZ2 was extracted from the Protein Data Bank (PDB entry code: 3CYY). For the long peptide and the phosphorylated peptide bound systems, the three upstream residues upstream were built using DSViewerPro 5.0. The added residues extend along the N-terminal end of the short peptide, the conformation of which is in accordance with the dominant conformation observed in the full length carboxyl terminal domain of Cx43 (PDBID: 1R5S) [37]. Hydrogen atoms were added using GROMACS 4.0 package. The complex was solvated in a 8.0×8.3×8.0 nm^3^ rectangular periodic box by water molecules represented by simple point charge (SPC) model [38]. The system was then neutralized by Na^+^ or Cl^−^ counter ions.

### MD simulations

MD simulations were carried out using GROMACS (version 4.0.5) software package [39], [40] and Gromos96 (version 43A2) force field [41], [42]. Each system was first minimized with steepest descent algorithm by 5000 steps and l-bfgs algorithm by 5000 steps with all the heavy atoms restrained. The resulted system was then minimized without any constraints, to eliminate any unfavorable contacts. After the relaxation process, each system was gradually heated from 0 K to 300 K in 60 ps, followed by constant temperature equilibration at 300 K for 30 ps. Then, 80 ns simulations were carried out in the NPT ensemble, where the temperature (300K) and pressure (1 atm) were maintained by Berendsen temperature and pressure coupling method [43]. All bonds that contain hydrogen atoms was constrained by Lincs algorithm [44], allowing a 2 fs time integration step. Nonbonded interactions were treated by a 12-Å cutoff. The electrostatic interactions were calculated by the particle-mesh Ewald (PME) method [45]. The potential and forces in between grid points were computed with a grid spacing of 0.12 nm and a fourth-order B-spline interpolation. The non-bonded pair-list cutoff was set to 0.9 nm and the pair-list was updated every 5 time steps.

### Cluster Analysis

Cluster analysis was used to study different peptide conformations sampled in the simulations. The RMSD-based GROMOS algorithm [46] was used for the analysis of the MD trajectories. Before clustering, the conformations of the peptides in the two binding sites of PDZ2 dimer in both trajectories were combined to make full use of the simulation data. The conformations in the trajectories were picked every four frames, resulting in totally 40004 conformations. Then a least root mean square deviation (RMSD) fit was performed based on the backbone atoms of the last three residues at the C-terminal end (position 0, −1 and −2) of the Cx43 peptides. In the clustering process, the difference between two structures was evaluated by the RMSD of the backbone atoms of the whole Cx43 peptide and the cutoff value was set to 1.5 Å.

### Free energy calculation

The free energy changes due to the phosphorylation at Ser(-9) of Cx43 peptide was calculated from the thermodynamics cycle shown in Figure S1. Dual topology method [47] was employed to perform these “alchemical transformation”. Phosphorylation transformations of peptide-bound and unbound states were performed using free energy perturbation (FEP) method [48], in which the Hamiltonian is made as a function of a coupling parameter λ that varies progressively from 0 to 1 corresponding to N nonphysical intermediates connecting the two thermodynamic states (i.e. wild type and phosphorylated states). The free energy difference between the two states can be expressed as
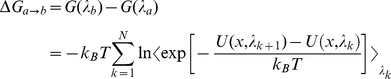
(1)where *k_B_* is the Boltzmann constant, *T* is the temperature, and *U(x;λ_k_)* is the potential energy of the system that dependent on the Cartesian coordinates and the coupling parameter *λ_k_*. As the 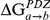 is zero, 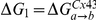. In the course of the alchemy transformations, the reaction paths were divided into 118 and 69 windows in the complex and free peptide systems respectively with uneven widths from λ = 0 to 1. Narrower windows were defined toward the end points of the simulation to avoid singularities. Each window involved 80 or 110 ps of equilibration in peptide or complex systems respectively, followed by 100 ps of data collection, summing up to a total of 12 or 22 ns simulations. The absolute free energy of phosphorylation involves the alchemy transformations of both a side chain (Ser to pSer) and two sodium counterions (the charge of each from 0 to +1) in order to maintain the overall charges of the system being zero throughout the transformation. Assuming that each free energy difference computed at a given *λ_k_*-state constitutes an independent observable, the error was determined using a first-order approximation, in which the change in the Gibbs free energy between two intermediate states is expressed as:
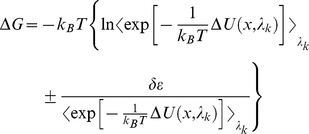
(2)

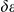
 is the statistical error of the ensemble average, 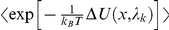, defined as:
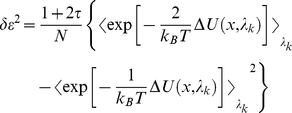
(3)where *N* is the number of the samples accrued in the free energy calculation, and (1+2*τ*) is the sampling ratio of the latter.

## Supporting Information

Figure S1 **Thermodynamic cycle used to calculate the difference of binding free energy between wild type and pSer(-9) systems (see text for more details).**
(DOC)

Table S1 **The average dihedral angles that describe the inter-domain orientation along the MD simulation trajectories.**
(DOCX)
