# Supplementary material for: Detailed Regulatory Mechanism of the Interaction between ZO-1 PDZ2 and Connexin43 Revealed by MD Simulations
Source: PLoS One. 2011 Jun 23;6(6):e21527. doi: 10.1371/journal.pone.0021527 (PMC3121883; doi:10.1371/journal.pone.0021527)

**Figure S1**: Thermodynamic cycle used to calculate the difference of binding free energy between wild type and pSer(-9) systems (see text for more details)


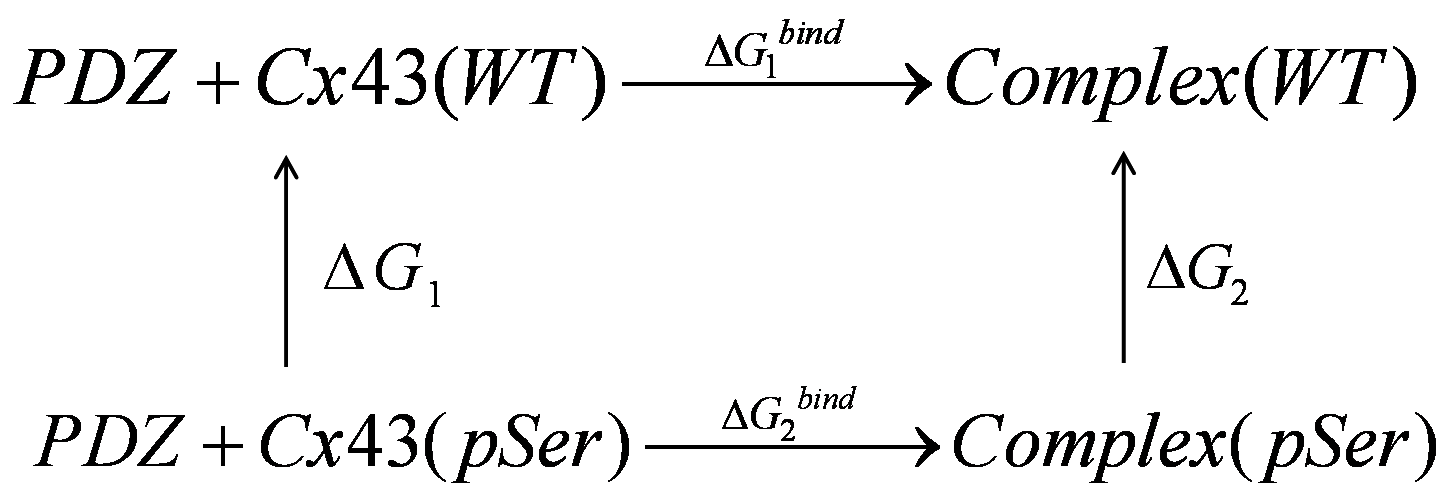

Supplement: Figure S1 — Thermodynamic cycle used to calculate the difference of binding free energy between wild type and pSer(-9) systems (see text for more details). (DOC) [file pone.0021527.s001.doc]
