# Supplementary material for: Detailed Regulatory Mechanism of the Interaction between ZO-1 PDZ2 and Connexin43 Revealed by MD Simulations
Source: PLoS One. 2011 Jun 23;6(6):e21527. doi: 10.1371/journal.pone.0021527 (PMC3121883; doi:10.1371/journal.pone.0021527)
Supplement: Table S1 — The average dihedral angles that describe the inter-domain orientation along the MD simulation trajectories. (DOCX) [file pone.0021527.s002.docx]

Table S1. The average dihedral angles that describe the inter-domain orientation along the MD simulation trajectories ^a^

|  | Average dihedral angle (degree) | Standard deviation  (degree) |
| --- | --- | --- |
| Apo (1^st^ run) | 152.9 | 6.5 |
| Apo (2^nd^ run) | 164.0 | 9.4 |
| Short form (1^st^ run) | 175.0 | 14.4 |
| Short form (2^nd^ run) | 149.2 | 9.7 |
| Long form (1^st^ run) | 181.3 | 8.5 |
| Long form (2^nd^ run) | 172.0 | 8.6 |
| pSer (1^st^ run) | 185.7 | 6.3 |
| pSer (2^nd^ run) | 154.7 | 8.9 |

^a^The definition of the dihedral angle is described in the text.
